# Retinal Organoids Long-Term Functional Characterization Using Two-Photon Fluorescence Lifetime and Hyperspectral Microscopy

**DOI:** 10.3389/fncel.2021.796903

**Published:** 2021-12-10

**Authors:** Yuntian Xue, Andrew W. Browne, William C. Tang, Jeffrey Delgado, Bryce T. McLelland, Gabriel Nistor, Jacqueline T. Chen, Kaylee Chew, Nicolas Lee, Hans S. Keirstead, Magdalene J. Seiler

**Affiliations:** ^1^Department of Biomedical Engineering, University of California, Irvine, Irvine, CA, United States; ^2^Stem Cell Research Center, University of California, Irvine, Irvine, CA, United States; ^3^Department of Ophthalmology, Gavin Herbert Eye Institute, University of California, Irvine, Irvine, CA, United States; ^4^Institute for Clinical and Translational Science, University of California, Irvine, Irvine, CA, United States; ^5^AIVITA Biomedical, Inc., Irvine, CA, United States; ^6^Department of Physics and Astronomy, University of California, Irvine, Irvine, CA, United States; ^7^Department of Physical Medicine & Rehabilitation, University of California, Irvine, Irvine, CA, United States; ^8^Department of Anatomy & Neurobiology, University of California, Irvine, Irvine, CA, United States

**Keywords:** human embryonic stem cell, retinal organoids, two-photon microscopy, fluorescence lifetime imaging, hyperspectral imaging, functional imaging, single cell RNA sequencing

## Abstract

Pluripotent stem cell-derived organoid technologies have opened avenues to preclinical basic science research, drug discovery, and transplantation therapy in organ systems. Stem cell-derived organoids follow a time course similar to species-specific organ gestation *in vivo*. However, heterogeneous tissue yields, and subjective tissue selection reduce the repeatability of organoid-based scientific experiments and clinical studies. To improve the quality control of organoids, we introduced a live imaging technique based on two-photon microscopy to non-invasively monitor and characterize retinal organoids’ (RtOgs’) long-term development. Fluorescence lifetime imaging microscopy (FLIM) was used to monitor the metabolic trajectory, and hyperspectral imaging was applied to characterize structural and molecular changes. We further validated the live imaging experimental results with endpoint biological tests, including quantitative polymerase chain reaction (qPCR), single-cell RNA sequencing, and immunohistochemistry. With FLIM results, we analyzed the free/bound nicotinamide adenine dinucleotide (f/b NADH) ratio of the imaged regions and found that there was a metabolic shift from glycolysis to oxidative phosphorylation. This shift occurred between the second and third months of differentiation. The total metabolic activity shifted slightly back toward glycolysis between the third and fourth months and stayed relatively stable between the fourth and sixth months. Consistency in organoid development among cell lines and production lots was examined. Molecular analysis showed that retinal progenitor genes were expressed in all groups between days 51 and 159. Photoreceptor gene expression emerged around the second month of differentiation, which corresponded to the shift in the f/b NADH ratio. RtOgs between 3 and 6 months of differentiation exhibited photoreceptor gene expression levels that were between the native human fetal and adult retina gene expression levels. The occurrence of cone opsin expression (OPN1 SW and OPN1 LW) indicated the maturation of photoreceptors in the fourth month of differentiation, which was consistent with the stabilized level of f/b NADH ratio starting from 4 months. Endpoint single-cell RNA and immunohistology data showed that the cellular compositions and lamination of RtOgs at different developmental stages followed those *in vivo*.

## Introduction

With the increase in life expectancy and the resulting aging population, blindness has become a global health problem. Age-related macular degeneration (AMD) is the most common retinal disease among people over 60 years old in the United States (Thapa et al., 2020). There is a lack of effective drug therapy for most retinal diseases. In recent decades, stem cell-based therapies are showing promises as effective treatments (Singh and Nasonkin, 2020). Human embryonic stem cells (hESCs) or induced pluripotent stem cells (iPSCs) derived retinal organoids (RtOgs) are self-organized tissues that recapitulate *in vivo* retinal development (Nakano et al., 2012; Zhong et al., 2014; Wahlin et al., 2017; Fligor et al., 2018). RtOgs exhibit similar structures and cell types as *in vivo* retina, and are used for many applications, including drug screening (Llonch et al., 2018), disease modeling, developmental biological research (Bell et al., 2020), and transplantation therapies (Shirai et al., 2016; McLelland et al., 2018; Lin et al., 2020; Thomas et al., 2021).

However, a significant obstacle in RtOg research is the lack of techniques to non-invasively monitor developmental progress and to perform quality control. Currently, most researchers applied immunostaining techniques (Maity et al., 2013; Shannon et al., 2020) to identify protein distribution and expression within RtOgs. While immunostaining has high specificity and can highlight the different structures in the tissue with amplified signals (Shannon et al., 2020), the main drawback of this technique is that it requires the tissues to be fixed, which are then no longer available for further studies. Due to the high heterogeneity in the RtOg culturing techniques and the resulting RtOg quality (Mellough et al., 2019), a more reliable approach with consistent outcomes is needed for live organoid characterization.

In recent years, 2-photon microscopy (2PM), which is an imaging technique that functions with fluorescence and pulsing laser (Sanderson et al., 2014), has become an alternative for single-photon imaging due to its reduced phototoxicity and photodamaging effects on the imaged tissues (Palczewska et al., 2010) while offering higher imaging resolution (Rubart, 2004; Bush et al., 2007). A 2PM utilizes two photons to simultaneously excite fluorophores in the tissues, reducing the energy per photon by half compared to single-photon microscopy. Lower energy deposited in the specimens extends the limit of imaging duration without adversely affecting the viability of the tissues under examination due to photodamage (Bastiaens and Squire, 1999). Furthermore, 2PM uses red or infrared laser sources, allowing better penetration depth (Belluscio, 2005) for use in ophthalmological research (Wei et al., 2021). Finally, native fluorophores intrinsic within cells can be excited in this wavelength range eliminating the need to introduce extrinsic fluorophores. This label-free live imaging eliminates errors stemming from non-specific binding of external fluorescent dyes (Datta et al., 2020).

In 2PM applications, two advanced modalities are used for live organ imaging: fluorescence lifetime imaging microscopy (FLIM) and hyperspectral imaging (HSpec) (Supplementary Figure 1). FLIM is commonly used to observe the metabolic states of live samples (Ranawat et al., 2019). Briefly, an impinging photon excites a molecule to a higher potential state. While returning to its ground state, the molecule emits fluorescence light at an intensity that decays over time. The lifetime of this fluorophore emission depends on its molecular environment regardless of the fluorophore concentration (Becker, 2012). Intrinsic fluorophores such as auto-fluorescent metabolic coenzymes nicotinamide adenine dinucleotide (NADH) and flavin adenine dinucleotide (FAD) are commonly targeted in FLIM (Cao et al., 2020). The spatial distribution of fluorescence is imaged with a charge-coupled device (CCD) camera (Lakowicz et al., 1992). Currently, FLIM has been used to observe the intracellular environment of single cells (Nakabayashi et al., 2017) and RtOgs (Browne et al., 2017). When FLIM is integrated with an ophthalmoscope (fluorescence lifetime imaging ophthalmoscope, FLIO), it can be used to help with retinal disease diagnoses (Dysli et al., 2017; Sauer et al., 2018).

One effective way to analyze FLIM imaging results is the phasor approach (Digman et al., 2008). In particular, the distribution map of the fluorescence lifetime of a sample or specimen represents the lifetime signature (Datta et al., 2015). NADH exhibits a shorter decay time in its free form in solution (0.4 ns) than when bound to lactate dehydrogenase (3.4 ns) (Ranjit et al., 2019). The ratio of free/bound NADH can be represented on a phasor plot to show their linear relationship to quantify the metabolic state of a specimen (Supplementary Figure 1D; Becker, 2012). Free NADH indicates glycolysis and a more proliferative state (stem cell-like), while bound NADH is correlated with more oxidative phosphorylation and a more differentiated state (Stringari et al., 2012b).

Hyperspectral imaging (HSpec), on the other hand, collects fluorescence spectral data associated with each pixel composing an image (Govender et al., 2007). Each pixel is therefore decomposed to multiple wavelength components and the spectral composition of a pixel is correlated with its chemical composition. HSpec generates higher-resolution three-dimensional datasets than multispectral imaging and thus capable of discerning distinct chemical species (such as retinol and retinoic acid) through overlapping spectra (Gao and Smith, 2015) and phasor approach (Supplementary Figure 1E; Browne et al., 2017). The phasor approach to analyzing hyperspectral data facilitates data decomposition by mapping complicated spectra to a 2-dimensional phasor plot by using a pair of Fourier sine and cosine transforms. Each pixel in the fluorescence image is mapped specifically to a location of the phasor plot by way of a determined phase monitor and angular position (Hedde et al., 2021). HSpec has been applied to imaging human retinal pigment epithelium (RPE) *ex vivo* to identify specific spectral signatures (Ben Ami et al., 2016). It has also been used *in vivo* to measure oxygen saturation in human retina (Dwight et al., 2016) and to discern potential Alzheimer’s disease biomarkers (Hadoux et al., 2019).

In this study, we applied 2PM to non-invasively examine the metabolic and structural changes in RtOgs long-term development. RtOgs derived from two stem cell lines were investigated with 2PM. In FLIM imaging we focused on the metabolic signatures indicated by free and bound NADH. In HSpec imaging we primarily investigated retinol, which is one of the retinoids produced in the visual cycle (Choi et al., 2021). The accumulation of retinol is a marker of functional photoreceptor cells (Browne et al., 2017). We further validated the functional imaging results with endpoint qPCR, single-cell RNA sequencing (sRNA-seq), and immunohistology.

## Materials and Methods

### Stem Cell Culture and Retinal Organoid Differentiation

The stem cell culture and RtOg initiation procedures were detailed in our previous publication (Xue et al., 2021), which were based on RtOgs differentiated from two hESC lines [cell line CSC14 with NIH registration no. 0284 and H9 (WA09) CRX-GFP with NIH registration no. 0062 (Collin et al., 2016)]. In the present study, we focused on CSC14-derived RtOgs in 10 GMP-compatible batches to perform long-term functional imaging and qPCR analyses. Subsequently, selected RtOgs were used for sRNA-seq and immunohistology analyses.

In addition, long-term imaging data from RtOgs differentiated from CRX-GFP hESCs were used in repeatability tests.

### Two-Photon Fluorescence Lifetime Microscopy and Hyperspectral Imaging

A Zeiss LSM 780 microscope (Carl Zeiss, Jena, Germany) equipped with a multi-photon laser source at 740 nm (Spectra-Physics Mai Tai, Mountain View, CA, United States) was used to perform both FLIM and HSpec 2P imaging through a Plan-Apochromat 20×/0.8 M27 objective (Carl Zeiss).

Fluorescence lifetime imaging microscopy imaging settings used in this study were the same as in our previous publication (Xue et al., 2021). Before imaging, the system was calibrated on frequency factor and lifetime with coumarin 6 solution, which has the known lifetime of 2.5 ns. Briefly, imaging settings used were as follow: 256 × 256 frame size, 1.66 μm pixel size, 25.21 μs pixel dwell time and 8-bit pixel depth. Emission laser was collected by the photomultiplier tube (H7422p-40, Hamamatsu, Japan) and a320 FastFLIM FLIMbox (ISS, Champaign, IL, United States). Fluorescence emission photons were counted. The lifetime information of each pixel was extracted according to the intensity decay curve (Supplementary Figure 1B). Using Fourier transform, the lifetime information of each pixel was mapped to a phasor plot, which contained on the so-called universal circle. Each point on the boundary of the universal circle represents a single exponential lifetime of one type of molecule and the proportion between molecules followed linear relationships. In this study we focused on the lifetime of free (0.4 ns) and lactate bound (3.4 ns) NADH (Supplementary Figure 1D). Additional details for FLIM imaging and data and data analysis using the phasor approach with SimFCS software (Globals Software G-SOFT Inc., Champaign, IL, United States) have been published previously (Digman et al., 2008; Xue et al., 2021). The fraction of free and bound NADH was normalized to the orthogonal intersection value on the metabolic NADH trajectory line by mapping the phasor plot center of mass directly to the free-bound NADH axis. As demonstrated on the phasor diagram (Supplementary Figure 1D), F1 is the fraction of free NADH and F2 is the fraction of bound NADH. Free/bound ratio equals to F1/F2. To overlay the metabolic color map on the structural image of the RtOg, we assigned a color bar along the free and bound NADH line and assigned a color value to each pixel in the image depending upon its location on the phasor plot as shown in Supplementary Figure 1D.

Similarly, HSpec imaging settings were detailed in our previous publication (Browne et al., 2017). Briefly, the fluorescence emission spectrum of 410 to 690 nm was collected with a 32-channel detector. The spectrum information of each pixel (Supplementary Figure 1C) was transformed into the data point on the spectral phasor plot (Supplementary Figure 1E). On the phasor plot different molecules have their unique “fingerprints.” By using the HSpec image analytical software (Translational Imaging Center, University of Southern California) (Cutrale et al., 2013, 2017), we were able to circle out the region that retinol located on the phasor plot and recolor that portion of pixels back on the HSpec image, thus the retinol distribution could be visualized.

### Quantitative Polymerase Chain Reaction (qPCR) Analysis

The primers for qPCR test are listed in Supplementary Table 1 (Qiagen, Germantown, MD, United States). In total, 14 retinal progenitor and photoreceptor genes and one housekeeping gene used as reference gene were examined for gene expression profile. Human fetal (HFE, age 137 days = 4.6 months) and adult retinal (HA) tissue (Eye bank, UCI-20-153-C-T) were used as positive controls. RtOgs aged from day 51 to 159 were grouped according to similar differentiation stages. Each sample set consisted of 3∼5 RtOgs and there were at least three samples in each group. Trizol reagent (Qiagen), DNase I digestion (Invitrogen, TURBO, Waltham, MA, United States), and phenol-chloroform extraction (Fisher) were used to isolate RNA and an RT2 cDNA synthesis kit (Qiagen) was used to synthesize cDNA. RT2 SYBR Green with ROX qPCR master mix (Qiagen) was used for amplification, which was performed with the following protocol: 95°C (15 min), 40 cycles at 95°C (15 s each), 55°C (30 s each), and 72°C (30 s each). The annealing temperature was 60°C. The double delta cycle threshold (Ct) method was used to calculate the fold expression with day 0 undifferentiated hESC (line CSC14) as the negative control. For analysis and heatmap generation, non-detected amplification in the control tissue and RtOgs were assigned cycle threshold values of 40. Heat maps with values in log_2_ (Fold Expression) were generated using Graphpad Prism software (Graphpad Software LLC, La Jolla, CA, United States).

### Single-Cell RNA Sequencing

To further compare the change of the cellular type of our RtOgs, we chose two typical time points – Day 57 and day 172 – for single-cell RNA sequencing analysis. RtOgs around D57 are in a multipotent state and this corresponds with the RtOg age when they are used in transplantation studies because their differentiation and proliferation potential allows integration with host tissue. In our previous publications we used RtOgs between 30 and 70 days of differentiation for transplantation in the retinal degenerate rat models and observed vision improvement (McLelland et al., 2018; Lin et al., 2020). The other time point D171 corresponds with fully matured RtOgs when photoreceptor cells with outer segment-like structures are present.

A total of 14 RtOgs on Day 57 (D57) and 12 RtOgs on D171 were dissociated using papain-based enzymatic digestion (Worthington papain dissociation NJ, United States). Cell viability was tested with 0.4% trypan blue in a hemocytometer (>90%). The concentration was adjusted to ∼870 live cells/μl for day 171 samples and ∼822 cells/μl for day 57 ones. The samples were prepared for scRNA-seq library within 5 min. The cell dissociation, library preparation, and data analysis were detailed in Xue et al. (2021). The raw data were uploaded to ArrayExpress and the accession number is E-MTAB-11121.

### Immunohistology

Retinal organoids on D118 and D169 of differentiation were fixed with cold 4% paraformaldehyde in 0.1 M Na-phosphate buffer for 1 h, cryoprotected (30% sucrose), and frozen in optimum cutting temperature (OCT) compound (PolarStat Plus, StatLab, McKinney, TX, United States). They were then cryo-sectioned into 10 μm serial sections and stored at –20°C. The primary and secondary antibodies used are listed in Supplementary Table 2. The staining procedure was detailed in Xue et al. (2021). Fluorescent sections were imaged with a Zeiss LSM700 confocal microscope (Zeiss, Oberkochen, Germany). ImageJ software (NIH, United States) was used for cell counting.

## Results

### Functional Imaging Revealed Retinal Organoids Long-Term Metabolic and Structural Development

**FIGURE 1:**
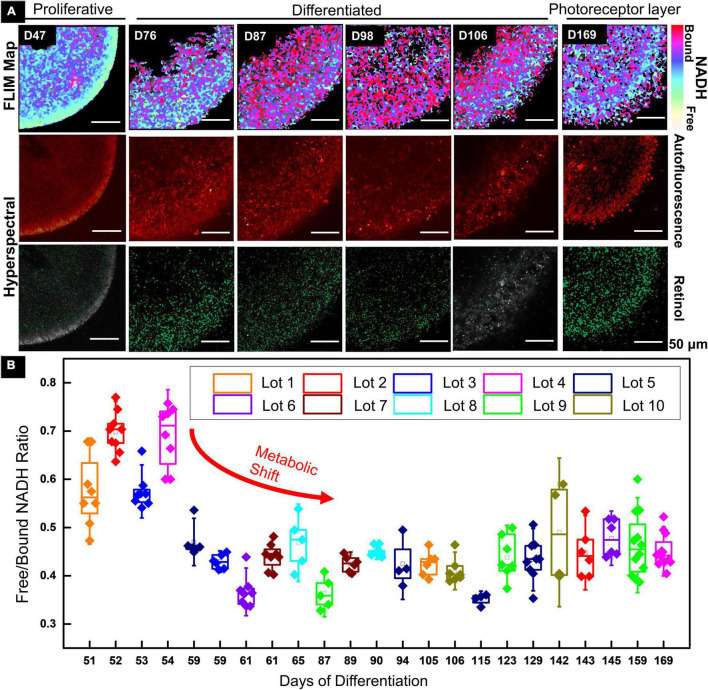
Functional imaging results of CSC-14 hESCs derived RtOgs. **(A)** FLIM and HSpec images of a typical RtOg. RtOg ages were from D47 to D169 of differentiation. The first row shows the pseudo-color-coded images that indicated free/bound (f/b) NADH ratio distribution. The second row shows the total autofluorescence emission from all intrinsic fluorophores that were excited by a 740 nm laser in HSpec scanning mode. The third row shows the retinol distribution in the imaged cross section and the results were calculated with spectral phasor plots from HSpec images. **(B)** f/b NADH ratio boxplot that summarized RtOgs from D51 to D169 of differentiation. The data set included 10 GMP-compatible batches of RtOgs. The boxplot indicates the 25th percentile, median and 75th percentile of the datapoints and the error bar indicates 1.5× standard deviation.

A 2PM functional imaging was performed on the 10 GMP-compatible batches from CSC-14 hESCs derived RtOgs. Figure 1A shows the results of representative RtOgs in long-term culture. The FLIM maps showed the spatial distributions of metabolic activities within a section of the RtOgs, confirming overall cellular viability and revealing a long-term developmental trend. RtOgs before D59 showed a higher f/b NADH ratio (Figure 1B) than the more mature ones. The FLIM map at D47 showed a more glycolytic surface with higher proliferative activities and the HSpec retinol distribution spread in the inner layer (Figure 1A, first column). As the RtOgs progressed through differentiation, a decrease in f/b NADH ratio was observed between D54 and D115 (Figure 1B). This was consistent with the false-color FLIM maps showing a shift to a red-dominated profile indicating a more differentiated state (Figure 1A, second to fourth columns). However, it was observed that the total metabolic activities partially shifted back toward glycolysis around D120 as shown in the gradual rise in the f/b NADH ratios, which then settled on a value slightly higher than the minimum (Figure 1B). At the fully matured stage (D169), the presence of outer segment-like structure in the photoreceptor layer was observed, and the outer nuclear layer exhibited a glycolytic surface (Figure 1A, sixth column). The HSpec autofluorescence image also showed a compact outer nuclear layer and a denser retinol distribution.

**FIGURE 2:**
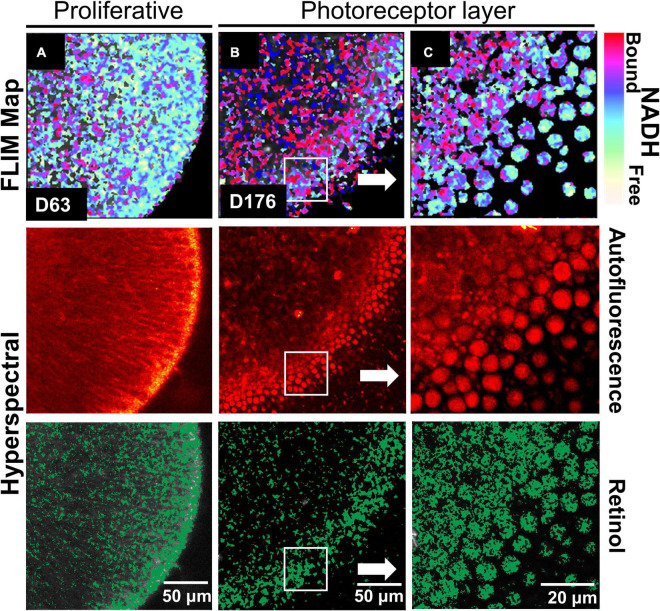
Functional imaging results of CRX-GFP hESCs derived RtOgs. FLIM and HSpec images of a typical RtOg are shown. RtOgs on **(A)** D63 and **(B)** D176 of differentiation. **(C)** The magnified view of the outer surface of the RtOg on D176.

We investigated RtOgs differentiated from a second stem cell line (CRX-GFP hESCs) and performed similar functional imaging. A similar metabolic trend was observed throughout the RtOg development. As shown in Figure 2A, the RtOg at a young age (D63) exhibited a larger area of the glycolytic surface in the FLIM map consistent with its proliferative activities than on D176, when only the outermost layer showed elevated glycolytic activities (Figures 2B,C), which was the metabolic signature of photoreceptor cells (Browne et al., 2017). The HSpec autofluorescence image showed a more distinct layering over time. The retinol also accumulated in the region where photoreceptors were located.

In summary, FLIM demonstrated retinal organoid development with a metabolic transformation from predominantly glycolytic to predominantly oxidative which occurred between 2 and 3 months of culture.

### Molecular Analyses Validated the Developmental Changes Shown in Functional Imaging

**FIGURE 3:**
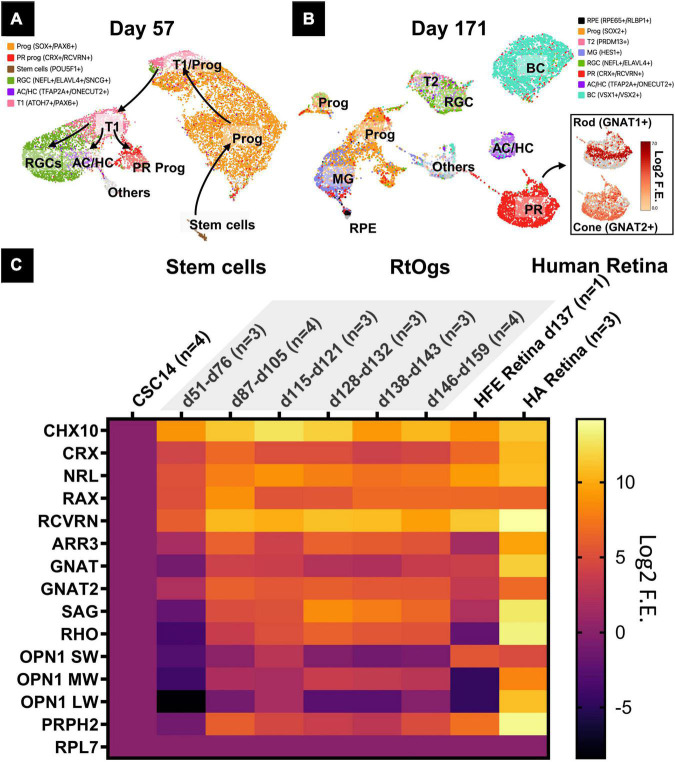
Gene profiles of RtOgs at different ages. **(A,B)** sRNA-seq UMAP showing the cell types in young (D57) and mature (D171) RtOgs. **(C)** qPCR heatmaps of RtOgs at various differentiation stages. CSC-14 hESCs derived RtOgs (negative control) were grouped according to similar day ranges. RPL7 was the housekeeping gene used for reference. Human fetal retina (HFE) and adult retina (HA) were used as positive control. Log_2_ F.E. – Log_2_ (Fold Expression). Cell legends in **(A,B)** Prog – retinal progenitor cell; RGC – retinal ganglion cell; PR prog – photoreceptor progenitor cell; T1 – cell in transition phase 1; AC/HC – amacrine cells and horizontal cells; BC – bipolar cells; T2 – cell in transition phase 2; PR – photoreceptor cell; RPE – retinal pigment epithelium cell.

Retinal organoids of two different stages (D57 and D171) were analyzed with sRNA-seq and the cell clusters were identified in the UMAP based on previous studies (Figures 3A,B; Shekhar et al., 2016; Sridhar et al., 2020). The young age group on D57 consisted of mainly retinal progenitor cells (51%), retinal ganglion cells (21%), cells in transition phase 1 (12%), and photoreceptor progenitor cells (7%) (Supplementary Table 3). On D171, additional cell types were observed with retinal progenitor cells (24%), bipolar cells (21%), and photoreceptor cells (20%) (Supplementary Table 4). Among photoreceptors, 25% were rods and 55% cones (Supplementary Table 4).

Retinal progenitor and photoreceptor marker genes were found with qPCR in RtOgs between D51 and D159 (Figure 3C). The stem cell group was used as negative control and human retina groups (fetal and adult) as positive control. The data showed that: (1) retinal progenitor genes were expressed in all groups; (2) mature photoreceptor genes were expressed after 2 months of differentiation consistent with the time point when a shift in f/b NADH ratio in long-term imaging (Figure 3B); (3) RtOgs more than 3-month old (D87-D159) exhibited a photoreceptor gene expression level between that of human fetal retina (HFE) and human adult retina (HA); and (4) The RtOgs at 4 months of differentiation showed more OPN1 SW and OPN1 LW (cone opsins) indicating the start of maturation, which was consistent with the f/b NADH ratio settling on a stable value from 4 months and onward (Figure 3B).

### 2PM Versus Immunohistology on Photoreceptor Imaging

**FIGURE 4:**
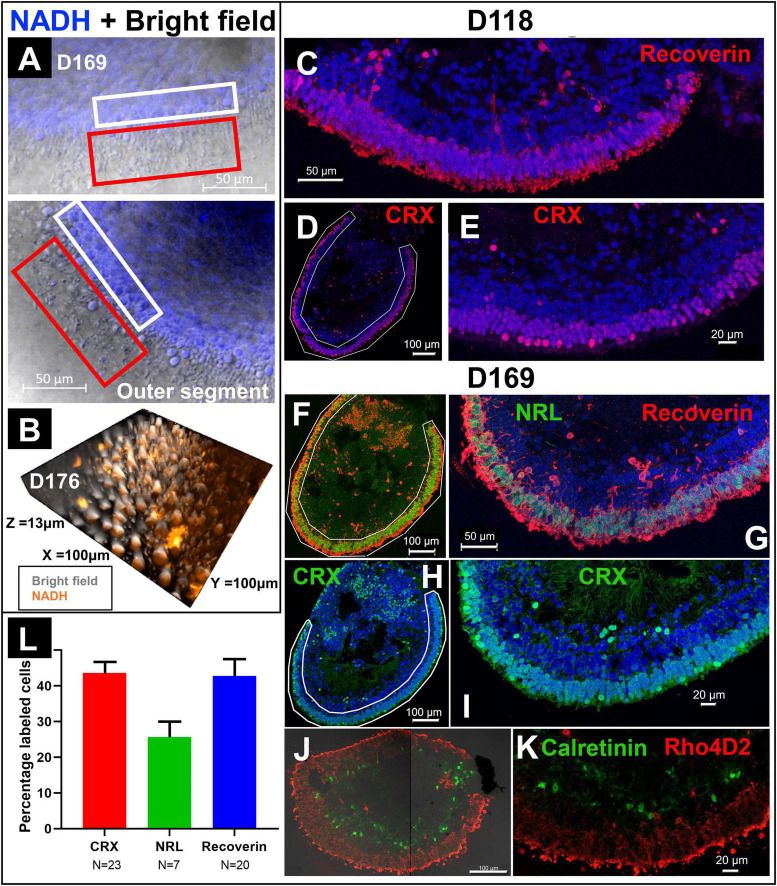
Comparison of immunohistology and 2P autofluorescence imaging. **(A)** Live imaging of a RtOg on D169 of differentiation using 2P microscopy (combination of brightfield and NADH autofluorescence). The white box frames the outer nuclear layer (ONL) and the red box the outer segment-like structures. **(B)** 3-D reconstruction from Z-stack images of the surface of a RtOg on D176 with 2P microscopy (combination of brightfield and NADH autofluorescence). **(C–E)** Immunohistology images of a RtOg at D118. **(F–K)** Immunohistology images of a RtOg on D169. **(L)** Cell counting plot from selected immunohistology slides (Error bar is the standard error of the mean). Antibody-marked cells: Recoverin and CRX – photoreceptor cells; NRL – rod photoreceptor cells; Rho4D2 – rod photoreceptor cells; Calretinin – amacrine cells. Nucleus were stained with DAPI (blue).

In addition to FLIM and HSpec, 2PM also provided high-resolution brightfield images. Figure 4A showed the combination of brightfield and NADH autofluorescence images of the cross section of a RtOg. The outer segment structures were fully preserved and clearly shown. The 3D representation of the organoid surface as shown in Figure 4B was reconstructed from Z-stack images. The 2PM images indicated that NADH autofluorescence was mainly distributed in the outer nuclear region. Immunohistology on the same organoid (Figures 4F–K) provided further details on the cell types and laminated structures. However, the outer segment structures were not fully preserved after the sectioning steps necessary for performing immunohistology.

Young RtOgs that preserved proliferative capability and multipotency have expressed more retinal progenitor marker based on our previous studies (McLelland et al., 2018; Lin et al., 2020). The RtOgs fully matured in around 4 months of differentiation. Immunohistology showed that RtOg on D118 developed CRX + and recoverin + photoreceptor layer (Figures 4C–E). In addition, RtOgs on both D118 and D169 showed distinct inner and outer nuclear layers. The cell counting results from immunohistology slides showed >40% CRX and recoverin + cells in the outer retinal rim. The percentage of NRL + cells (rod-specific marker) was lower, indicating a high percentage of cones in the organoids (Figure 4L). This result was consistent with the sRNA-seq findings that cone photoreceptors were more abundant than rods.

## Discussion

The functional imaging results in our work showed the different developmental stages in RtOg progression that were consistent with published literature (Browne et al., 2017; Xue et al., 2021). Stem cells are known to be glycolytic (green/yellow color coding for high f/b NADH ratio). As they differentiate, their metabolic activities progress toward more oxidative phosphorylation (red/purple color coding for low f/b NADH ratio) (Stringari et al., 2012a; Wright et al., 2012). As RtOgs mature, their outermost surface develops photoreceptors with a glycolytic signature and retinol accumulation consistent with prior observations (Browne et al., 2017). These time-dependent metabolic signatures are powerful indicators in determining and predicting RtOg differentiation stages.

The scRNA-seq data offered a comprehensive profile of RtOgs’ cell type in early and mature differentiation stages. Metabolic imaging indicated that RtOgs were more proliferative in the early stage, confirming scRNA-seq and qPCR data that more progenitor cell markers were expressed. Further, FLIM also showed that the shift to a more differentiated stage started between the 2nd to the 3rd month and stabilized in the 4th month and afterward. The gene expression profile by qPCR also demonstrated this trend because mature photoreceptor genes were gradually expressed after 2 months of differentiation. However, although higher than human fetal retina, the matured photoreceptor gene expression level of the RtOgs after 4 months was still not comparable to human adult retina, which is one of the intrinsic limitations of *in vitro* organ differentiation.

Compared with conventional RtOg characterization methods such as immunohistology and qPCR, 2PM has the outstanding advantage of non-invasive live tissue imaging. In see section “Molecular Analyses Validated the Developmental Changes Shown in Functional Imaging,” we showed that 2PM was superior in examining the outer segment structures than immunohistology, which required preparation procedures including fixation, wash, and microtome that destroyed most of the delicate outer structures. Thus, in immunohistology, only a few slides sectioned at certain orientations showed partial outer segment structures. Further, 2PM FLIM and HSpec can also recapitulate the laminar structures on the RtOg surface at the cellular and molecular levels that are comparable to those obtained from immunohistology. Most importantly, 2PM approaches significantly reduce photodamage, allowing non-destructive RtOg characterization.

However, RtOgs in this study showed tissue heterogeneity initially and as differentiation progressed beyond 3 months. As shown in Figure 1B, the variations in f/b NADH ratios were higher during both early and late stages. To ensure analysis uniformity and consistency, we only chose the outer surface of each organoid to image and analyze the f/b NADH ratio. While the gene expression and scRNA-seq validated that photoreceptor cells were differentiated, not all organoids were able to develop an uniform and laminated photoreceptor layer on their outer surface, and the thickness of the laminar structure also varied, especially in the stages that organoids rapidly proliferated or differentiated. Further, only a few RtOg samples in the 4–6-month age range showed outer segment structures. In addition to biological heterogeneity intrinsic to developing RtOgs, the error from manual maintenance of retinal tissue in suspended dish culture may also cause a visible morphological difference among organoids in the 6 months range. To address this issue, we are developing an automated long-term culture bioreactor for nearly labor-free RtOg maintenance (Xue et al., 2021). When optimized and integrated with 2PM imaging system, the automated bioreactor can potentially increase imaging efficiency and allow scaled-up process and characterization of RtOg production. On this matter, 2PM is also a promising non-invasive method to evaluate the consistency of RtOgs quality differentiated from different culture protocols.

Beyond RtOg characterization, 2PM can be developed further as a tool for *in vivo* examination on animal models transplanted with RtOgs. Previous studies showed its applicability in mouse (Palczewska et al., 2014a) and primate (Palczewska et al., 2014b) models. The optimized conditions can be achieved by testing and adjusting the laser power (Alexander et al., 2016) and temporal specifications (Palczewska et al., 2020).

In addition to the two imaging modalities introduced in this study, there are other non-invasive methodologies can be applied to organoid research. Browne et al. has used optical coherence tomography (OCT) to image live RtOgs and found a reflectivity difference on the RtOgs’ surface (Browne et al., 2017). Furthermore, Scholler et al. (2020) developed a dynamic full-field OCT system to image live RtOgs and provided 3-dimensional color images that reflected organelle motility with sub-micrometer spatial resolution and millisecond temporal resolution. Dutta et al. (2020) used vis/near-infrared (NIR) spectroscopy to study the neurodevelopment of brain organoids. Recently, Hedde et al. (2021) implemented the sine/cosine snapshot phasor-based hyperspectral imaging method to image zebrafish retina and organelles, and significantly improved imaging speed when working together with light sheet microscopy. Overall, non-invasive imaging technologies are inevitably rising as valuable tools to investigate structural and functional biology.

## Conclusion

We have demonstrated a 2PM-based non-invasive imaging technique to monitor RtOg metabolic and structural changes at the cellular level throughout the entire differentiation and development process. The long-term functional imaging data showed that RtOgs from different cell lines and different batches exhibited a repeatable and predictable metabolic developmental process from more proliferative at an early stage to more differentiated at a later stage. The metabolic signature stabilized after 4 months, which was consistent with the time point in gene expression profile stabilization. The methodology and the findings of this study are of great value in live RtOgs characterization and monitoring, offering a potentially powerful tool in screening and quality control for RtOg production.

## Data Availability Statement

The original contributions presented in the study are publicly available. This data can be found here: The data are available on the ArrayExpress website under http://www.ebi.ac.uk/arrayexpress/experiments/E-MTAB-11121.

## Author Contributions

MS, AB, WT, GN, HK, and YX: concept and design. MS, AB, GN, HK, and WT: financial support, administrative support, and provision of study material. YX, JD, and BM: collection and assembly of data. YX, JD, AB, JC, KC, and NL: data analysis and interpretation. YX, MS, AB, WT, JC, KC, NL, BM, and JD: manuscript writing. YX, AB, WT, JD, BM, GN, JC, KC, NL, HK, and MS: final approval of manuscript. All authors contributed to the article and approved the submitted version.

## Conflict of Interest

MS has a patent from Ocular Transplantation LLC; BM is employed by AIVITA Biomedical; GN is employed by AIVITA Biomedical; HK is CEO and board member of AIVITA Biomedical. The remaining authors declare that the research was conducted in the absence of any commercial or financial relationships that could be construed as a potential conflict of interest.

## Publisher’s Note

All claims expressed in this article are solely those of the authors and do not necessarily represent those of their affiliated organizations, or those of the publisher, the editors and the reviewers. Any product that may be evaluated in this article, or claim that may be made by its manufacturer, is not guaranteed or endorsed by the publisher.
