# Supplementary material for: Retinal Organoids Long-Term Functional Characterization Using Two-Photon Fluorescence Lifetime and Hyperspectral Microscopy
Source: Front Cell Neurosci. 2021 Dec 10;15:796903. doi: 10.3389/fncel.2021.796903 (PMC8707055; doi:10.3389/fncel.2021.796903)
Supplement: Supplementary file 1 [file Data_Sheet_1.docx]

**
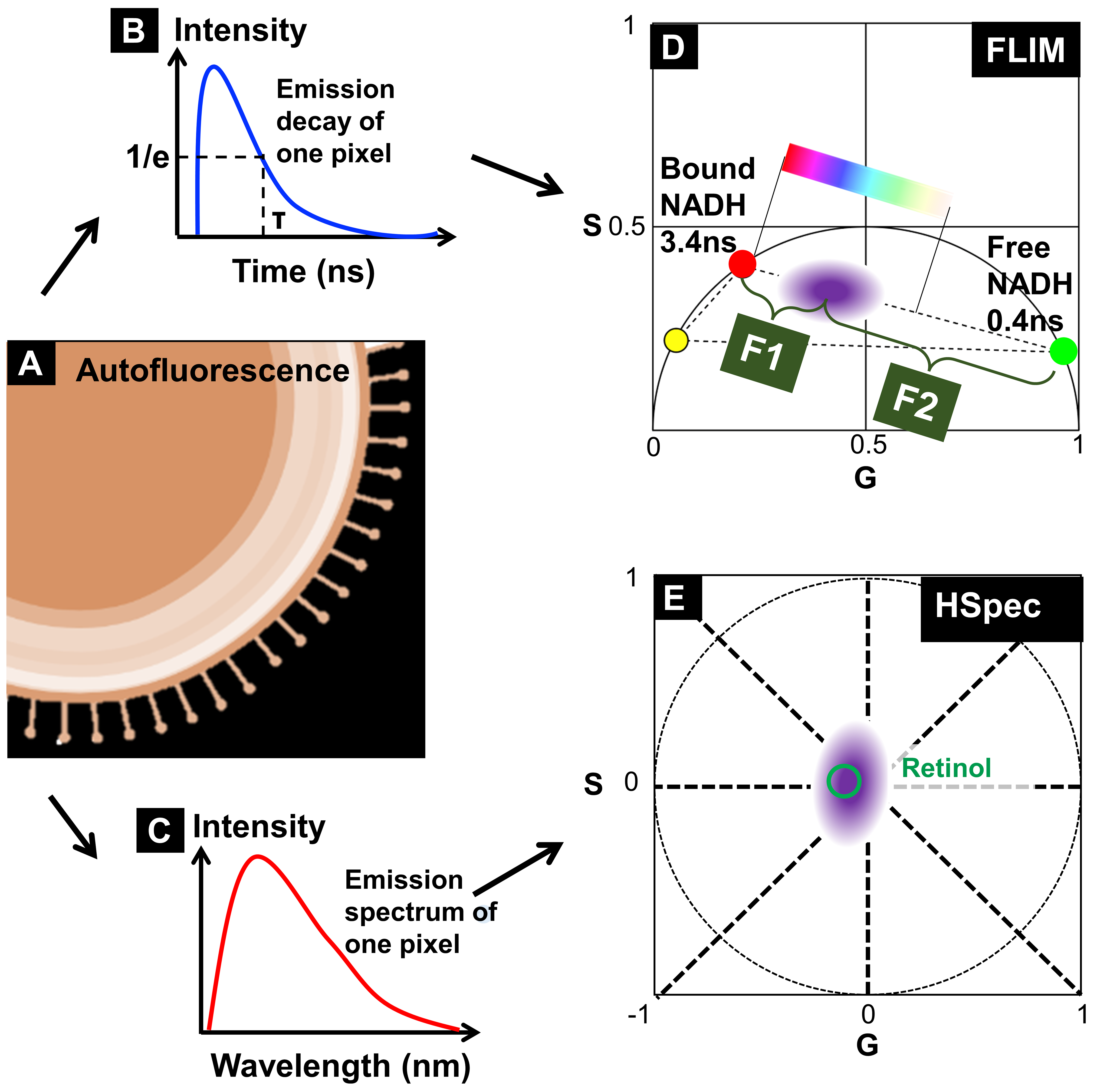
**

**Figure S1:** FLIM and HSpec techniques used in this study. (**A**) Schematic of an autofluorescence intensity map of a live RtOg’s cross section generated by 740 nm pulse laser excitation composed of pixels and each pixel was analyzed to generate an emission intensity decay curve (**B**) and the spectrum curve (**C**). The lifetime τ is the time point when the intensity decreased to 1/e of the peak autofluorescence intensity (**B**). The lifetime (**B**) and spectrum (**C**) curve were then transformed mathematically to two a lifetime and hyperspectral phasor plot. (**D**) Phasor plot of FLIM showed a 2D diagram for intrinsic fluorophores with different lifetimes (bound NADH is indicative of oxidative phosphorylation and free NADH represents glycolysis). On the phasor plot components followed a linear relationship, thus, the fractions of free and bound NADH were F1 and F2, respectively. The free/bound ratio was obtained by calculating F1/F2. (**E**) Phasor plot of hyperspectral imaging showed the distribution of the intrinsic retinol fluorophore located within the point cloud representing the hyperspectral phasor analysis.

**Table S1: Information of qPCR primers**

| Gene name | Official full name | GeneGlobe ID |
| --- | --- | --- |
| CRX | Cone-rod homeobox | QT01192632 |
| CHX10 (VSX2) | Visual system homeobox 2 | QT00221081 |
| NRL | Neural retina leucine zipper | QT01005165 |
| RAX | Retina and anterior neural fold homeobox | QT00212667 |
| RCVRN | Recoverin | QT00014098 |
| ARR3 | Arrestin 3 | QT00000182 |
| SAG | S-antigen visual arrestin | QT01007958 |
| PRPH2 | Peripherin 2 | QT00094094 |
| GNAT | G-protein subunit alpha transducin | QT00235606 |
| GNAT2 | G-protein subunit alpha transducin 2 | QT00008764 |
| RHO | Rhodopsin | QT01017058 |
| OPN1SW | Opsin 1, short wave sensitive | QT00017304 |
| OPN1MW | Opsin 1, medium wave sensitive | QT00040887 |
| OPN1LW | Opsin 1, long wave sensitive | QT01007356 |
| RPL7 | Ribosomal protein L7 | QT01670137 |

**Table S2: Information of Antibodies**

| Antibody | Species | Concentration | Manufacturer | Catalog # | RRID |
| --- | --- | --- | --- | --- | --- |
| Rhodopsin (Rho4D2) | Mouse | 1:100 | Gift of Dr. Molday [1], University of British Columbia | N/A | AB_2315273  AB_2315274 |
| Human NRL | Goat | 1:100 | R&D Systems | AF2945 | AB_2155098 |
| Recoverin | Rabbit | 1:2000 | Millipore | AB5585 | AB_2253622 |
| Calretinin | Goat | 1:100 | Novus | AF5065 | AB_2068516 |
| CRX | Rabbit | 1:100 | Biorbyt | orb192904 | AB_2810291 |

**Table S3: scRNA seq cell type and percentage – Day 57**

| Cell type | Cluster gene | Cell # | Cell % |
| --- | --- | --- | --- |
| Retinal progenitors | SOX2+, PAX6+ | 5434 | 51% |
| Photoreceptors progenitors | CRX+, RCVRN+ | 786 | 7% |
| Stem cells | POU5F1 | 157 | 1% |
| Retinal ganglion cells | NEFL+, ELAVL4+, SNCG+ | 2216 | 21% |
| Amacrine cells/ Horizontal cells | TFAP2A+, ONECUT2+ | 179 | 2% |
| T1 phase | ATOH7+, PAX6+ | 1265 | 12% |

**Table S4: scRNA seq cell type and percentage – Day 171**

| Cell type | Cluster gene | Cell # | Cell % |
| --- | --- | --- | --- |
| Retinal progenitors | SOX2+ | 2480 | 24% |
| RPE | RPE65, RLBP1+ | 174 | 2% |
| Muller glia | HES1+ | 986 | 9% |
| Retinal ganglion cells | NEFL+, ELAVL4+ | 794 | 8% |
| Amacrine cells/ Horizontal cells | TFAP2A+, ONECUT2+ | 332 | 3% |
| Bipolar cells | VSX1+, VSX2+ | 2212 | 21% |
| T2 phase | PRDM13+ | 358 | 3% |
| Photoreceptors | CRX+, RCVRN+ | 2078 | 20% |
| Matured Rods (among Photoreceptors) | GNAT1+ | 560 | 25% |
| Matured Cones (among Photoreceptors) | GNAT2+ | 1148 | 55% |

**Reference**

1. Molday, R.S. and D. MacKenzie, *Monoclonal antibodies to rhodopsin: characterization, cross-reactivity, and application as structural probes.* Biochemistry, 1983. **22**(3): p. 653-60.
